# 

**Published:** 1892-04

**Authors:** 


*American Veterinary College,*

*New York.*

APRIL, 1892.

### CONTENTS:

ORIGINAL ARTICLES: PAGE

Strongylus Rubidus, a New Species of Nematode, Parasitic in Pigs. By Albert Hassall and C. W. Stiles,........ 207

Navicular Disease. By William Bryden, V. S........ 210

A Bacterial Disease of Animals. By Henry G. Pyle, D. V. S........ 215

Parasites. By Cooper Curtice, M. D........ 223

The Use of Lithium in Veterinary Practice. By S. S. Baker, D.V.S........ 236

Lump Jaw—Continued. By R. W. Hickman, V. M. D........ 239

Sciatica. By John Scott........ 242

Is Osteoporosis Infectious? By J. C. McNeil, V. M. D........ 244

SELECTIONS:

Ear Vertigo in the Horse, Induced by Aspergillus Nigricans. By Thos. B. Goodall, F. R. C. V. S........ 247

EDITORIAL:

Apology........ 251
American Veterinary College—Commencement........ 252
New York College of Veterinary Surgeons—Commencement........ 253
Chicago Veterinary College—Commencement........ 254
Faculty of Comparative Medicine—McGill University—Commencement........ 255
Ontario Veterinary College—Commencement........ 258

SOCIETY PROCEEDINGS:

California State Veterinary Medical Association........ 261
Massachusetts Veterinary Medical Association........ 261
Illinois State Veterinary Medical Association........ 263
Pennsylvania State Veterinary Medical Association........ 264
Kansas Veterinary Medical Association........ 267

REVIEWS:

Moeller's Surgery........ 267

Books and Pamphlets Received........ 268

